# Superhydrophobic Modification of *Sansevieria trifasciata* Natural Fibres: A Promising Reinforcement for Wood Plastic Composites

**DOI:** 10.3390/polym15030594

**Published:** 2023-01-24

**Authors:** Yanzur Mohd Aref, Rizafizah Othaman, Farah Hannan Anuar, Ku Zarina Ku Ahmad, Azizah Baharum

**Affiliations:** 1 Department of Chemical Sciences, Faculty of Science and Technology, University Kebangsaan Malaysia, UKM Bangi, Bangi 43600, Malaysia; 2 Polymer Research Centre, Faculty of Science and Technology, University Kebangsaan Malaysia, UKM Bangi, Bangi 43600, Malaysia; 3 Department of Mechanical Engineering, Universiti Pertahanan Nasional Malaysia, Kem Sungai Besi, Kuala Lumpur 57000, Malaysia

**Keywords:** superhydrophobic, natural fibres, treatment, silica nanoparticles, fluorosilane, *Sansevieria trifasciata*

## Abstract

*Sansevieria trifasciata* fibre (STF) is a lignocellulosic material which could be utilised for reinforcement composites. Surface modification is often needed to improve the compatibility of hydrophilic STF and hydrophobic resin. In this study, treatments for natural fibres to attain superhydrophobic properties were carried out using silica nanoparticles and fluorosilane. *Sansevieria trifasciata* fibres (STF) were subjected to treatment by deposition of silica (SiO_2_) nanoparticles which were prepared by the sol-gel method, then followed by modification with fluorosilane, namely 1H, 1H, 2H, 2H-perfluorooctyltriethoxysilane (PFOTS). The presence of SiO_2_ nanoparticles and PFOTS were evaluated using Fourier transform infrared spectroscopy (FTIR) and scanning electron microscopy with energy dispersive X-ray spectroscopy (SEM-EDX). The crystallisation properties and thermal behaviour of STF were studied through X-ray diffraction (XRD) and thermogravimetric (TGA) analysis, respectively. The hydrophobicity of STF was determined by water contact angle (WCA) measurement. The results show that nanoscale SiO_2_ particles were deposited on the STF surface, and PFOTS were covalently linked to them. The SiO_2_ nanoparticles provide surface roughness to the fibres, whereas the long-chain fluorine on PFOTS lowered the surface free energy, and their combination in these treatments has successfully modified the STF surface from hydrophilic into superhydrophobic with a WCA of 150° and sliding angle of less than 10°. Altogether, a non-toxic, simple, and promising method of imparting hydrophobicity on natural fibres was developed, opening new opportunities for these fibres as reinforcement for composite parts.

## 1. Introduction

Increasing environmental awareness has recently stimulated interest in utilizing natural fibres as an alternative to artificial materials in many sectors, including composite manufacturing. By exploiting the benefits of natural fibres, such as low cost, lightweight, abundantly available, renewable, and biodegradable, natural fibres have overtaken synthetic fibres as reinforcement in polymer composites [1,2]. However, some issues remain, notably the hydrophilic properties of natural fibres, which allow them to absorb moisture from the surroundings, leading to poor interfacial bonding with the hydrophobic polymer matrices. This condition promotes the degradation of natural fibres and impairs the performance of the resulting composites [3]. Hence, imparting hydrophobicity on natural fibres using specific treatments is necessary to improve their compatibility with organic matrices and widen their application as reinforcement for composite parts.

Hydrophobicity depends on surface roughness and surface chemistry [4,5], where the combination of a rough micro–nanostructure and a low surface energy material could develop a superhydrophobic surface [6]. This concept was discovered from the superhydrophobic phenomena of lotus leaves. Their structure combines a waxy layer with low surface energy and a rough structure with protrusions [7]. The superhydrophobic surface has received great attention due to its excellent water repellency, and mimicking the so-called “lotus effects” could be an alternative to surface modification.

Many approaches have been implemented to provide the surface with nanoscale roughness, such as sol-gel processing, layer-by-layer assembly, flame synthesis coating, etching, and electrospinning [6,8,9,10]. Several research papers have reported these approaches and mostly covered substrates based on non-renewable materials. Some of those methods required specialized equipment, involved harsh conditions, and were only applicable to specific materials [11]. For example, the flame synthesis coating offers a simple and low-cost method to prepare a durable superhydrophobic surface; however, a specially designed heat conduction system with a controlled velocity of gas flow rate is required to form the desired nanoparticles [12]. Esmeryan et al. developed robust superhydrophobic carbon soot coatings using a specially designed cone-shaped aluminium chimney, decreasing the oxygen level used during combustion [13]. Another study by Esmeryan et al. demonstrated a novel method for producing ultradurable liquid-repellent soot coatings onto different fabrics using an optimized combustion system by means of a metal chimney [14]. Compared to these methods, sol-gel processing is a simpler way of producing a rough surface without using a specific set-up and conditions. It is more popular and widely used in cellulose-based materials due to its low-temperature procedure that could deposit the nanoparticle layer on the surface of substrates [15]. The nanoparticles produced could be either SiO_2_ or TiO_2_, but SiO_2_ is more common due to its low toxicity [16]. It is possible to create the nanoparticles coating with a size of 50–500 nm by the sol-gel method, which could provide a suitable roughness on the surface [17].

To exhibit superhydrophobicity, a chemical modification to lower the surface energy of the roughened surface is necessary. Some commonly used reactive molecules for low-surface-energy modification are organosilanes, fluoro silane, perfluorinated compounds, and polymers [18]. Among the most effective modifying agents are fluorosilane, and the most frequently used are fluoro-functional trichloro- and trialkoxysilanes [19,20]. Silane is a multifunctional molecule which acts as a coupling agent. Silane could modify the fibre surfaces to improve their wettability by reacting with functional groups on the fibre surface or making their crosslinking possible. One factor contributing to silane’s ability to generate a hydrophobic surface is its organic substitution [21]. Fluorinated hydrocarbon substituent contains the hydrophobic moiety that allows the silane to induce surface hydrophobicity due to the low surface energy of fluorine [12]. The fluorine/carbon atomic ratio is a key aspect of defining hydrophobicity. It has been demonstrated by Hsieh et al. from studies with carbon nanofibre arrays [22]. As expected, as more F atoms are introduced, lower surface energy is achieved. Hence, PFOTS, a fluorosilane with a long chain of fluorinated hydrocarbon substituents, is believed to provide high hydrophobicity to the natural fibres.

To date, many research papers covering different aspects of superhydrophobicity have been published. Various substrates have been fabricated with superhydrophobic surfaces, including glass, silicon wafers, and metal surfaces [23,24]. However, the case of lignocelluloses has proven to be more challenging, partly because of the influence of hydrophilic components in complex and highly hierarchical structures [12]. Efforts in developing superhydrophobic surfaces based on cellulose have been growing steadily in these past few years, for example, in wood, cotton fabrics, and paperboard [12,25], but there is limited research on pristine natural fibres.

This work aims to develop superhydrophobic natural fibre surfaces by describing a two-step treatment procedure. The natural fibre used was *Sansevieria trifasciata* fibres (STF) which have a high potential in composite manufacturing. The first step is roughening the STF surface by forming silica (SiO_2_) nanoparticles using the sol-gel process. The roughened surface is modified in the second step by the hydrophobic 1H, 1H, 2H, 2H-perfluorooctyltriethoxysilane (PFOTS). The expectation was that the surface morphology and chemistry of lotus leaves could be imitated, with the SiO_2_ nanoparticles in combination with the long chain fluorosilane on it and that the coating would lower the surface free-energy of the treated natural fibre.

## 2. Materials and Methods

### 2.1. Materials

**Figure 1:**
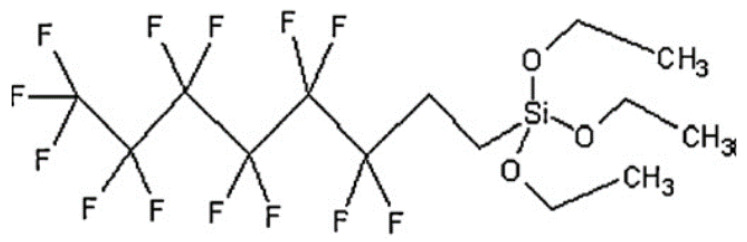
Molecular structure of 1H, 1H, 2H, 2H-perfluorooctyltriethoxysilane (PFOTS) [18].

*Sansevieria trifasciata* (ST) leaves were collected from the Bangi region. Sodium hydroxide (NaOH) with 95% purity, commercial grade, was purchased from Macron Fine Chemicals. Tetraethoxysilane (TEOS) with 98% purity and ammonium hydroxide (NH_4_OH) were purchased from Sigma Aldrich, St. Louis, MO, USA. 1H, 1H, 2H, 2H-perfluorooctyltriethoxysilane (PFOTS), as shown in Figure 1, with 98% purity, was purchased from Apollo Scientific, UK. Glacial acetic acid with AR grade was purchased from Systerm. Absolute ethanol with 99% purity was also obtained from Systerm.

### 2.2. Sansevieria trifasciata Fibres Preparation

Fresh ST leaves were thoroughly washed in running water to remove dust and other impurities from the surface. Then, they were cut to about 1.0–1.5 cm long and sun-dried for a week to ensure minimal water in the fibres. The dried ST leaves were ground with a grinder for 1 min to break them into fine particles. Finally, they were sieved with a 500 µm sieve to obtain the desired size of 500 µm STF.

### 2.3. Pre-Treatment

Raw STF from the previous preparation was immersed and stirred continuously in 4% wt NaOH solution at room temperature for 1 h. The weight ratio of NaOH solution per fibre content was fixed at 15:1. Next, the STF was washed using distilled water containing a few percentages of acetic acid to remove the alkali residue. Washing continued until no alkalinity was detected in the waste washing water using a pH meter. The fibres were filtered and then dried in an oven at 80 °C for 24 h.

### 2.4. SiO_2_-Mediated Hydrophobic Treatment

In this treatment, the ratio of STF to SiO_2_ solution used was 12:1. The SiO_2_ solution was prepared using the sol-gel method described by Stober et al. [26]. In this method, TEOS was hydrolysed in an ethanol solution with an NH_4_OH catalyst at room temperature to form SiO_2_ nanoparticles. We mixed 30 mL of NH_4_OH, 915 mL of absolute ethanol, and 135 mL of distilled water, and they were magnetically stirred for 15–30 min to produce a homogeneous solution. After that, 420 mL of TEOS was added to the solution while stirring, and the mixture was left to stir for 2 h at room temperature to produce SiO_2_ nanoparticles. The STF was then soaked in the prepared SiO_2_ solution and stirred continuously with a mechanical stirrer for 2 h at room temperature. Next, the STF was filtered and rinsed with distilled water until the rinse water was clear, and the pH was checked to ensure it was neutral. Finally, the SiO_2_-treated STF was obtained and dried in an oven at 80 °C overnight.

### 2.5. Fluorosilane Hydrophobic Treatment

**Figure 2:**
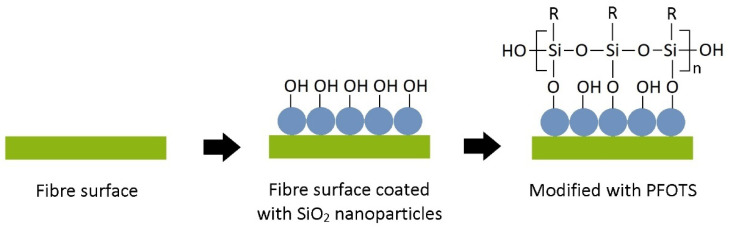
The schematic illusion of the treatment process to form a superhydrophobic STF surface. R represents the hydrophobic group of PFOTS: –CH_2_CH_2_(CF_2_)_5_CF_3_.

Hydrophobic treatment was performed on SiO_2_-treated STF by the chemical vapour deposition of 1H, 1H, 2H, and 2H-perfluorooctyltriethoxysilane (PFOTS). The fibre sample was placed in a sealed vessel with a smaller unsealed vessel containing about 0.3 mL of POTS. The sealed vessel was then put in an oven at 125 °C to enable the PFOTS vapour silane group to react with the hydroxide group on the STF surface. After 2 h, the sample was removed to another clean, sealed vessel and heated at 150 °C for another 2 h to volatilise the unreacted PFOTS molecules on the substrate. The formation process of the superhydrophobic fibre surface is illustrated schematically in Figure 2.

### 2.6. Characterization of STF

Chemical changes occurring on STF surfaces subjected to treatment were analysed with a Fourier transform infrared (FTIR) instrument (Spectrum GX, Perkin Elmer) equipped with an attenuated total reflection (ATR) accessory. Details: spectral range from 600 to 4000 cm^−1^, 64 scans, and resolution of 4 cm^−1^. The crystallinity of STF was studied using X-ray diffraction (XRD) analysis. A D8-Advance, Bruker AXS model of the XRD instrument equipped with CuKα monochromatic radiation (λ = 0.1539 nm) was used to measure the XRD pattern. The thermal decomposition behaviour of STF was analysed using a thermogravimetric analyser (TGA) (TGA/SDTA 85-F, Mettler Toledo). The samples were heated steadily at a rate of 5 °C/min from 30 to 600 °C in a nitrogen medium (20 mL/min). The surface morphology of STF and their corresponding elemental compositions were examined through a field emission scanning electron microscope (FE-SEM, Leo 1450VP) with a typically installed energy dispersive X-ray (EDX) spectroscopy attachment. The fibre was scattered and picked piece by piece to be placed under the microscope. A thin platinum (Pt) layer was spattered onto the sample surfaces to improve conductivity prior to observation. The wettability of untreated and treated STF was characterised by water contact angle (WCA) measurements using a goniometer (Holmarc model, combined with video equipment) at room temperature. For this analysis, the STF was pressed into pallet form with 0.3 cm thickness to obtain a flat and rigid sample of STF. This is because the WCA analysis needs to be performed on a flat solid sample. The sessile drop method was applied, and the WCA values were acquired 60 s after a water droplet of 5 μL was placed on the surface of fibre samples. More than five measurements were averaged to obtain one representative WCA value for each sample.

## 3. Results and Discussion

### 3.1. Chemical Changes on STF Surface

**Figure 3:**
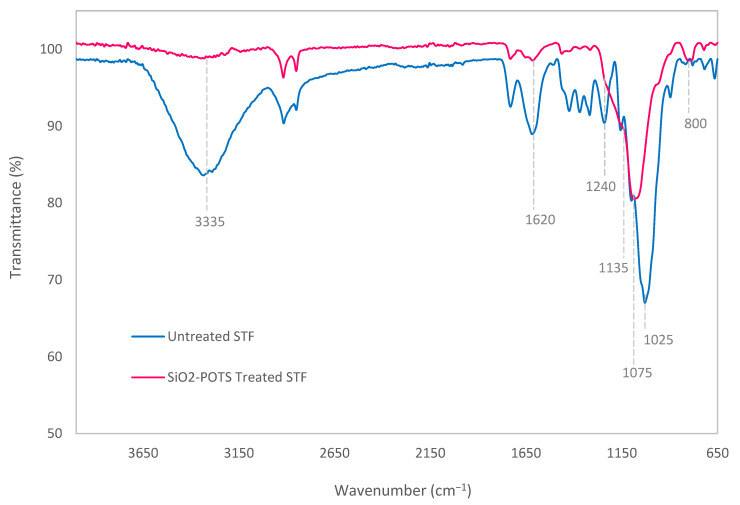
FTIR spectra of untreated and SiO_2_-PFOTS treated STF.

Fourier transform infrared (FTIR) spectroscopy was used to investigate the changes in the main chemical bonding of STF after surface modification. Figure 3 compares the FTIR spectra of the untreated STF and the one treated with SiO_2_ nanoparticles and PFOTS. For the untreated STF, the spectra show a prominent absorption band at 1025 cm^−1^ that corresponds to the C–O stretching of the ether group in cellulose. Whereas in the spectra of treated STF, this peak has slightly shifted to a higher wave number at around 1075 cm^−1^ due to the asymmetric stretching vibration of Si–O–Si bonds that partially overlapped with the C–O band [25,27]. This indicates the formation of Si–O–Si covalent bonds by the reaction between SiO_2_ nanoparticles and PFOTS molecules on the surface of treated STF. A weak peak at approximately 800 cm^−1^ that appeared after treatments is also assigned to the Si–O–Si band. It is associated with its symmetric stretching vibration [28,29]. Moreover, the C–F bands of PFOTS molecules are identified in the range of 1230–1135 cm^−1^ in the spectra of treated STF, where the peaks are not too prominent because they may be covered by the strong absorption peak of Si–O–Si asymmetric stretching vibration [18]. Another significant peak is the broad absorption band at 3335 cm^−1^ representing the –OH group of cellulose which could be seen clearly in the spectra of untreated STF. Interestingly, this peak is almost invisible after treatments, indicating that the –OH group was almost completely obliterated. Additionally, the bending vibration of the –OH group is also detected in both spectra at about 1620 cm^−1^, but its peak is weaker in the treated STF [30]. These results revealed that the treatments using SiO_2_ nanoparticles and PFOTS managed to decrease the hydrophilicity of STF.

### 3.2. Crystallization Properties of STF

**Figure 4:**
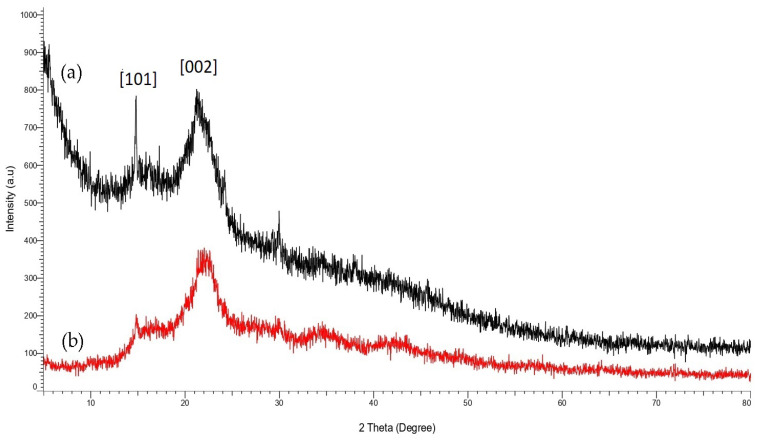
XRD pattern of (**a**) untreated STF and (**b**) SiO_2_-PFOTS treated STF.

X-Ray diffraction (XRD) characterisation was carried out to further understand the effect of SiO_2_-PFOTS treatments on STF crystallinity and physical structure. The obtained XRD patterns of untreated and treated STF are shown in Figure 4. Both curves exhibit two significant diffraction peaks commonly seen in STF [31]. The first peak observed at 2*θ* of approximately 15.5° belongs to the 101 lattice plane, indicating the amorphous elements of cellulose, hemicellulose, and lignin. The second peak at 2*θ* of approximately 22° is ascribed to the 002 plane representing the cellulose crystalline phase [32]. The peaks revealed that STF has a semi-crystalline structure which consists of both amorphous and crystalline phases. After the treatments with SiO_2_ nanoparticles and PFOTS, no structural transformation of cellulose occurred in STF. However, the amorphous peak of treated STF shows a significant reduction in intensity, probably due to the removal of some amorphous constituents from the fibre surface [33]. Thus, the amorphous removal resulted in the rearrangement of the fibres’ crystalline region, which leads to a better packing of cellulose chains and thus gives the fibres a more crystalline structure [34]. Hence, it can be deduced that the SiO_2_-POTS treatments increased the crystallinity of STF.

### 3.3. Thermal Decomposition Behaviour of STF

**Figure 5:**
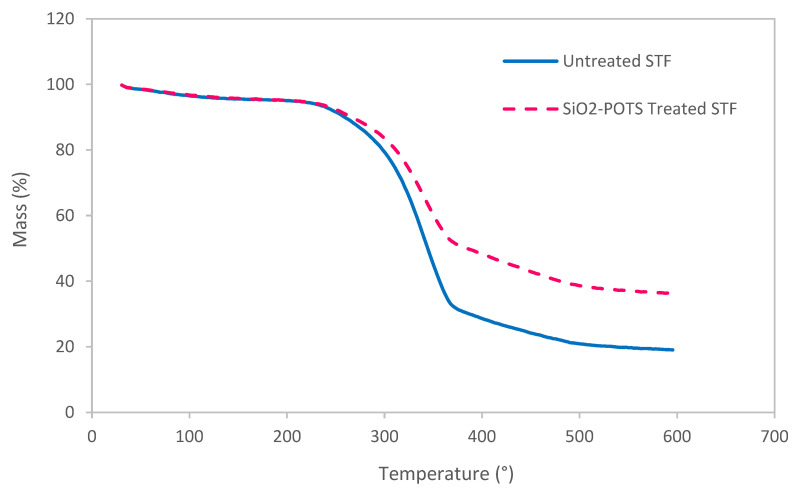
Thermogram of untreated and SiO_2_-PFOTS treated ST.

Thermogravimetric analysis (TGA) was performed to examine the thermal decomposition behaviour of ST fibres before and after surface treatments. Figure 5 illustrates the thermograms of both untreated and SiO_2_-PFOTS-treated STF. Three stages of mass losses observed in both curves represent the decomposition behaviour of STF at a specific temperature. The initial mass loss stage started in the temperature range of 30–100 °C, which refers to the release of moisture content by the fibres [35]. Then, the second decomposition stage occurs at 200–400 °C, with a major mass loss due to the main decomposition of hemicellulose, cellulose, and some lignin [36]. At this stage, it can be seen that the treated STF possess a lower mass loss compared to the untreated ones. This might be caused by decreased cellulose, hemicellulose, and lignin content in STF after the SiO_2_-PFOTS treatments, which made the fibres more hydrophobic [35]. For the final decomposition stage, the curve lies within 400–600 °C with approximately 7–14% mass loss indicating the decomposition of the lignin and other non-cellulosic materials [37]. At the endpoint after complete decomposition, a residual mass of about 19% and 36.2% were generated by the untreated and treated STF, respectively. These are residual masses from carbonaceous and inorganic materials that remain undecomposed at 600 °C [38]. A higher residual mass on treated STF is ascribed to the presence of SiO_2_ nanoparticles through treatments. The existence of these inorganic materials on fibres enhances their thermal stability, shown in the thermogram curve by the increase in the decomposition temperature of STF after the SiO_2_-PFOTS treatments [35].

### 3.4. Surface Morphology of STF

**Figure 6:**
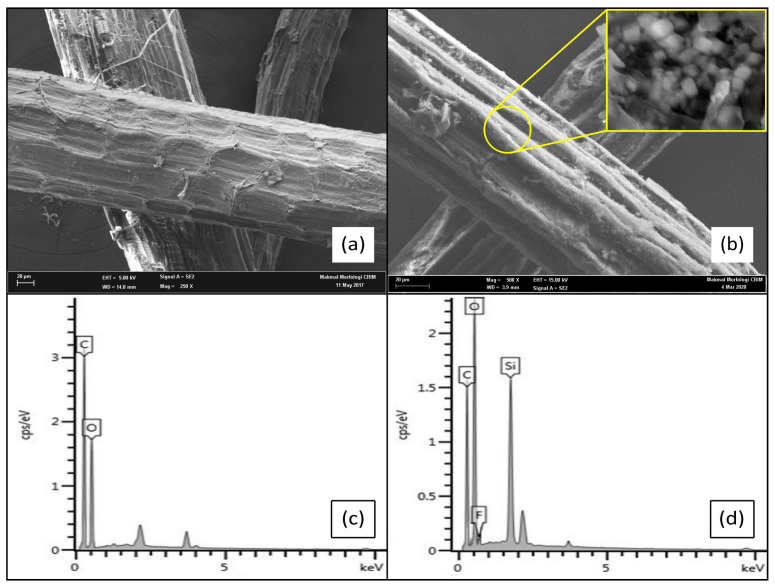
FE-SEM images and its corresponding EDX spectrum of (**a**,**c**) untreated STF (**b**,**d**) SiO_2_-PFOTS treated STF.

The field emission scanning electron microscopy (FE-SEM) images of STF before and after the SiO_2_-PFOTS treatments are presented in Figure 6a,b, respectively. The images are captured at 250× magnification and display that the surface of STF before treatment is relatively smooth compared to the surface after treatment. The rough surface of treated STF is attributed to the formation of SiO_2_ nanoparticles layer on their surfaces through the SiO_2_-PFOTS treatments. From this, we can deduce that the SiO_2_ nanoparticles have been successfully deposited on the surface of STF, thus providing the necessary roughness to create a superhydrophobic surface [25]. The image of treated STF in Figure 6b is further zoomed in to 5000× magnification, revealing the formation of SiO_2_ nanoparticles with a spherical shape. It could be observed that the fibre surface is densely packed with many irregular sphere particles of roughly 200–500 nm in diameter. The randomly distributed arrangement of silica spheres creates several gaps or voids between each other, thus roughening the surface of STF fibres [18]. To further confirm the presence of SiO_2_ nanoparticles and PFOTS, the electron dispersive X-ray (EDX) technique was also used in this SEM characterisation, where the elemental compositions on the STF surface were analysed. Figure 6c,d shows the EDX spectrum of untreated and treated STF, respectively. From Figure 6c, the C and O elements were found on the raw STF, referring to the components of fibres. When STF were treated with SiO_2_ nanoparticles and PFOTS, the Si and F elements appeared on the EDX spectrum, as shown in Figure 6d. The strong signal of Si elements indicates the existence of SiO_2_ nanoparticles and PFOTS combined on the STF surface through the treatments. The appearance of element F on the spectrum also belongs to PFOTS reagents and further proved its formation on the surface of STF.

### 3.5. Wetting Properties of STF

**Figure 7:**
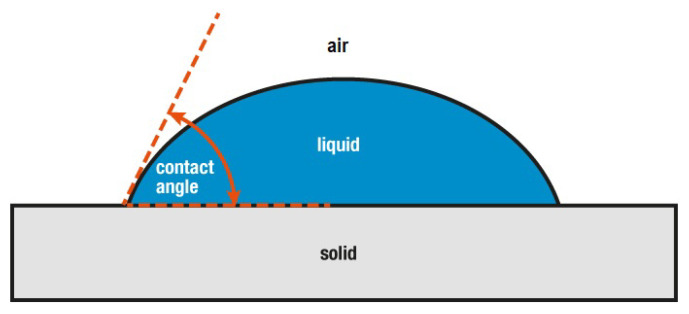
The water contact angle of a solid surface.

The surface wettability of STF before and after SiO_2_-PFOTS treatments was represented by its surface’s water contact angle (WCA). When a liquid drops on a flat, horizontal solid surface, an interaction occurs between the liquid, air, and the solid interface. The angle formed by the liquid droplet at the intersection across the three interfaces is referred to as the WCA (Figure 6) [39]. A surface with a high wettability, known as hydrophilic, has a WCA of less than 90°. Whereas, for a poor wettability surface known as hydrophobic, its WCA will be greater than 90°. A very high hydrophobic surface could reach a WCA of 150° and above, showing almost no contact between the water droplet and the surface. Such a surface is considered superhydrophobic [21]. Figure 7 shows the photographs of water droplets on the surface of STF where its WCA before and after each stage of treatment were compared.

**Figure 8:**
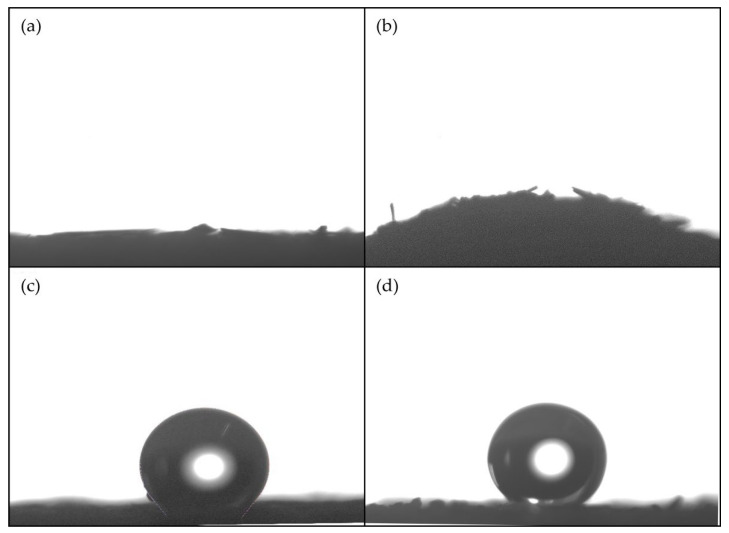
Images of water droplets on different surfaces: (**a**) untreated STF (**b**) SiO_2_ treated STF (**c**) PFOTS treated STF (**d**) SiO_2_-PFOTS treated STF.

Figure 8a demonstrates that the untreated STF is completely hydrophilic when the water droplet spreads out instantly into its surface, and the resulting WCA is 0°. The STF treated with only SiO_2_ in Figure 8b still shows a hydrophilic surface as it also absorbs the water droplet completely, and the WCA obtained is also 0°. This indicates the presence of abundant hydroxyl groups on the surface of SiO_2_ nanoparticles [40]. A slight bump observed on the photograph is due to the STF surface being wetted by the water droplet. In contrast, Figure 8c shows that after the treatment with only PFOTS, the STF surface turns hydrophobic by achieving a WCA of about 127°. Eventually, as seen in Figure 8d, a combination of SiO_2_ treatments followed by the PFOTS reaction resulted in a higher WCA of roughly 150° on the STF surface, which possesses superhydrophobic properties. The image of an almost fully round-shaped water droplet could be observed resting on top of the superhydrophobic STF surface without wetting it. To confirm its superhydrophobicity, the sliding angle (SA) was also checked, which is the critical inclination angle at which the substrate needs to be tilted until the droplet rolls off the surface [7]. The SA obtained was less than 10° and showed extremely high water-repellency, where water droplets bead up and freely roll off the surface without leaving any trace of the beads. These results show that the surface roughness of SiO_2_ nanoparticles, combined with the low surface energy of PFOTS, affected the wettability of the STFs surface.

The effectiveness of using PFOTS as a hydrophobic modifying agent was supported by comparing the WCA results obtained with a previous study that used a similar approach. For example, Wang et al. employed the same procedure of sol-gel coating on wood substrates followed by the hydrophobic modification, but with a different type of silane. A long-chain alkysilane, hexadecyltrimethoxysilane (HDMS), was used instead of fluorosilane. The results show that a highly hydrophobic wood surface was developed but did not reach superhydrophobicity, where the WCA obtained was 141° [25]. This WCA shows that compared to the long-chain alkylsilane of HDMS, PFOTS with long-chain fluorine could provide lower surface energy and better hydrophobicity.

### 3.6. Application and Performance of Superhydrophobic STF in Wood Plastic Composite

The modified STF surface, which changed from hydrophilic to superhydrophobic, has great potential as a reinforcement filler in wood plastic composites. This is because the superhydrophobic property of STF makes it more compatible with the hydrophobic polymer matrix in composite production. Hence, the mechanical properties of the resulting composite could be improved. One of the most widely used polymer matrices in wood plastic composites is polypropylene (PP) thermoplastic. The effect of STF treatments using SiO_2_ nanoparticles and PFOTS on the wettability and mechanical properties of 70/30% ST/PP composite was studied.

**Figure 9:**
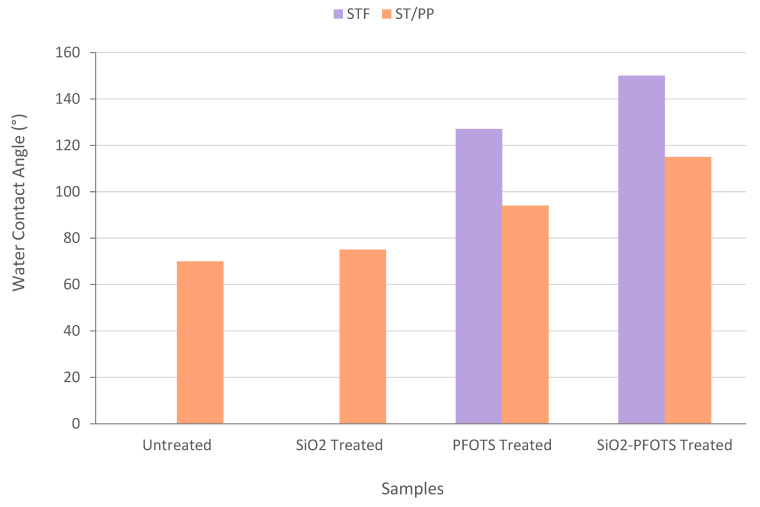
Comparison between the water contact angle of STF and ST/PP composite with different treatments.

Figure 9 compares the WCA of STF and ST/PP composites with different treatments. The graph clearly shows that for all PFOTS-treated and SiO_2_-PFOTS-treated samples, the WCA of both STF and ST/PP increases to more than 90°, showing that their surface has become hydrophobic. However, the WCA recorded by ST/PP composite is lower than STF for both PFOTS-treated and SiO_2_-PFOTS-treated samples. This may be caused by the lower WCA of PP (96°) than the PFOTS-treated and SiO_2_-PFOTS-treated STF, which slightly reduces the WCA of the resulting ST/PP composite. This explanation is in accordance with Jose et al., which stated that each material combined in a composite production would maintain its unique identity [41]. Besides, STFs are embedded in the resulting composite’s PP matrix, causing the STF surface to be covered by them [42]. Hence, the effect of surface roughness obtained by SiO_2_ nanoparticle treatment on the STF surface is no longer shown on the ST/PP composite. This is the reason why the WCA of the SiO_2_-PFOTS-treated ST/PP composite does not reach superhydrophobicity as obtained on the SiO_2_-PFOTS-treated STF. However, there is still an improvement from its original hydrophilic properties to become highly hydrophobic. SiO_2_-treated ST/PP wood composite PFOTS also have a more hydrophobic surface than treated samples with PFOTS only. This means fibre treatment that combines the roughness of SiO_2_ nanoparticles with the low surface energy PFOTS significantly produces a higher hydrophobic surface on wood composite.

**Figure 10:**
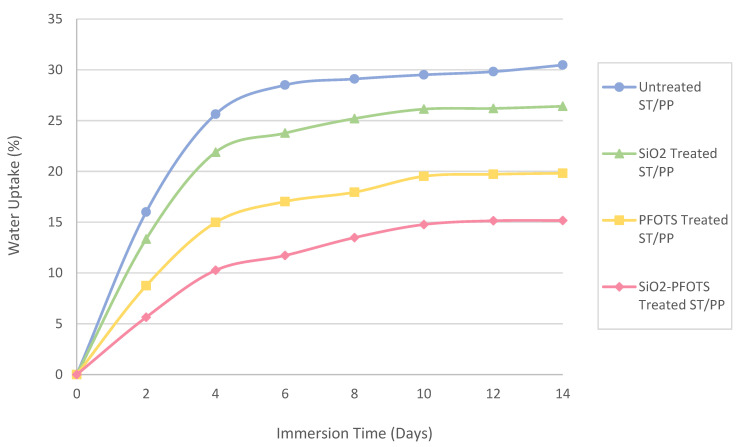
The water absorption rate of untreated ST/PP, SiO_2_ treated ST/PP, PFOTS treated ST/PP, and SiO_2_-PFOTS treated ST/PP.

The wettability of ST/PP composite is also evaluated by their water absorption rate. Figure 10 shows the water absorption behaviour of the ST/PP composite with different treatments. From the graph, the percentage of water uptake by each ST/PP composite increased with increasing immersion time and began to level off after the eighth day. The positive effect of STF modification can be observed when the percentage of water uptake by ST/PP composites decreases gradually after each treatment. The SiO_2_-PFOTS-treated ST/PP composite shows the most significant difference in water absorption rate compared to the other treatments. The largest reduction in water absorption rate with 50.21% water uptake is recorded by the SiO_2_-PFOTS treated ST/PP composite on the last day of the immersion period. It is followed by the PFOTS-treated ST/PP with s 34.95% reduction and SiO_2_-treated ST/PP with a 13.32% reduction. This observation is consistent with the WCA results, where the most hydrophobic ST/PP surface is treated with SiO_2_-PFOTS. Therefore, the STF treatment using SiO_2_ nanoparticles followed by PFOTS is confirmed to be the most suitable for producing wood–plastic composites with excellent anti-wetting properties.

**Figure 11:**
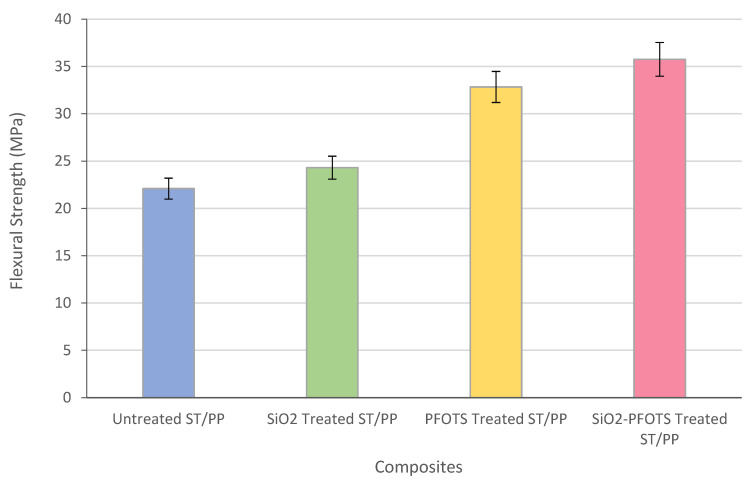
Flexural strength of untreated ST/PP, SiO_2_ treated ST/PP, PFOTS treated ST/PP, and SiO_2_-PFOTS treated ST/PP.

The mechanical property of 70/30% ST/PP wood–plastic composite was determined by its flexural strength. Figure 11 shows the flexural strength of ST/PP composites with different treatments. The graph shows that the flexural strength of ST/PP composites increases with each treatment of STF. This increasing flexural strength indicates that the surface modification of STF using SiO_2_ nanoparticles and PFOTS can improve the adhesion between STF and PP matrix in ST/PP composites. On the other hand, the untreated ST/PP composite has the lowest flexural strength due to poor compatibility between STF and PP. This matter is based on the statement of Abdullah and Ahmad (2012) in their study, where the flexural property shown by composites could explain the interaction and adhesion of the fibre–matrix interface in the composites [2]. The ST/PP composite with SiO_2_-PFOTS combined treatment shows the highest flexural strength compared to the other treatments that use only SiO_2_ nanoparticles or PFOTS. Compared to the untreated composite, the flexural strength value of SiO_2_-PFOTS treated ST/PP composite increases by 112%, from 22.09 MPa to 35.75 MPa. This most significant increment suggests that combining SiO_2_ nanoparticles with PFOTS treatment could improve fibre–matrix adhesion in ST/PP composite. This is because the STF surface is not only covered with the roughness of SiO_2_ nanoparticles, but the PFOTS-modified STF also formed covalent bonds between STF and PP, leading to noticeably stronger fibre–matrix adhesion results. Additionally, the combination of SiO_2_ surface roughness with the low surface energy of POFTS has given STF a superhydrophobic surface, which has improved its compatibility with the PP matrix [43].

Overall, we can say that the superhydrophobic coating on the fibre obtained in this work is comparable with similar results obtained by different technologies. The performance of superhydrophobic samples from two comparable references [44,45] is summarized in Table 1. The WCA obtained by our work and the other two studies does not show a big difference; however, our superhydrophobic fibre shows a more significant increase in its performance. The decreased performance of modified samples in compared studies may be attributed to the sensitivity of natural fibres to acid solutions used in their fabrication method. In contrast, the mild process used in our work leads to an enhanced performance of the superhydrophobic natural fibre.

## 4. Conclusions

In summary, a stable superhydrophobic surface has been successfully fabricated on STF by depositing SiO_2_ nanoparticles using a sol-gel process, followed by the modification using PFOTS. Eventually, a robust superhydrophobic STF surface with a WCA of 150° and sliding angle of less than 10° was obtained by combining the high surface roughness of SiO_2_ nanoparticles and the low surface free energy of PFOTS. These treatments provide a simple and non-toxic solution to make natural fibres superhydrophobic, thus opening new opportunities for these fibres to reinforce the composite parts.

## Figures and Tables

**Table 1:** Performance of reported superhydrophobic natural fibre surface fabricated via different methods.

Fabrication	Wettability	Mechanical Durability	Thermal Stability	References
SiO_2_ nanoparticles by sol-gel process, followed by modification with PFOTS	WCA = 150°, SA < 10°	Flexural strength increases from 22.09 MPa to 35.75 MPa	Main degradation temperature increases from 278 °C to 308 °C	This work
ZnO by the hydrothermal process followed by modification with stearic acid	WCA = 151°, SA < 5°	Breaking force decreases from 90 cN to 60 cN	Main degradation temperature decreases from 260 °C to 240 °C	[44]
PLA/CIN coating followed by nano-SiO_2_-modified stearic acid	WCA = 156.3° SA = N/A	Breaking elongation decreases from 3.5% to 3.1%	Main degradation temperature increases from 355 °C to 360 °C	[45]

## Data Availability

Not applicable.

## References

[1] Aref Y.M., Baharum A. (2018). Effect of fibre treatment using fluorosilane on *Sansevieria trifasciata*/polypropylene composite. Proceedings of the 2017 UKM FST Postgraduate Colloquium.

[2] Abdullah N.M., Ahmad I. (2012). Effect of chemical treatment on mechanical and water-sorption coconut fibre-unsaturated polyester from recycled PET. Int. Sch. Res. Netw..

[3] Ghasemi I., Farsi M. (2010). Interfacial behaviour of wood plastic composite: Effect of chemical treatment on wood fibres, Iran. Polym. J..

[4] Tan J.Y., Ang W.L., Mohammad A.W. (2021). Hydrophobic polyvinylidene fluoride membrane modified with silica nanoparticles and silane for succinic acid purification using osmotic distillation process. J. Kejuruter..

[5] Sajab M.S., Jauhari W.N.W.A.R., Chia C.H., Kaco H.Z.H., Noor A.M. (2018). Oleophilicity and oil-water separation by reduced graphene oxide grafted oil palm empty fruit bunch fibres. Sains Malays..

[6] Nakajima A., Hashimoto K., Watanabe T. (2001). Recent studies on super-hydrophobic films. Mon. Fuer Chem..

[7] Li S., Huang J., Chen Z., Chen G., Lai Y. (2017). A review on special wettability textiles: Theoretical models, fabrication technologies and multifunctional applications. J. Mater. Chem. A.

[8] Dorrer C., Rühe J. (2008). Some thoughts on superhydrophobic wetting. Soft Matter.

[9] Genzer J., Efimenko K. (2006). Recent developments in superhydrophobic surfaces and their relevance to marine fouling: A review. Biofouling.

[10] Stepien M., Saarinen J.J., Teisala H., Tuominen M., Aromaa M., Kuusipalo J., Mäkelä J.M., Toivakka M. (2011). Adjustable wettability of paperboard by liquid flame spray nanoparticle deposition. Appl. Surf. Sci..

[11] Levkin P.A., Svec F., Frechet J.M.J. (2009). Porous polymer coatings: A versatile approach to superhydrophobic surfaces. Adv. Funct. Mater..

[12] Song J., Rojas O.J. (2013). Approaching super-hydrophobicity from cellulosic materials. Nord. Pulp Pap. Res. J..

[13] Esmeryan K.D., Castano C.E., Bressler A.H., Abolghasemibizaki M., Mohammadi R. (2016). Rapid synthesis of inherently robust and stable superhydrophobic carbon soot coatings. Appl. Surf. Sci..

[14] Esmeryan K.D., Fedchenko Y.I., Gyoshev S.D., Lazarov Y., Chaushev T.A., Grakov T. (2022). On the development of ultradurable extremely water-repellent and oleophobic soot-based fabrics with direct relevance to sperm cryopreservation. ACS Appl. Biol. Mater..

[15] Bokov D., Jalil A.T., Chupradit S., Suksatan W., Ansari M.J., Shewael I.H., Valiev G.H., Kianfar E. (2021). Nanomaterial by sol-gel method: Synthesis and application. Adv. Mater. Sci. Eng..

[16] Mahltig B., Swaboda C., Roessler A., Böttcher H. (2008). Functionalising wood by nanosol application. J. Mater. Chem..

[17] Jalali R., Rezaei M., Nematollahi B., Baghalha M. (2019). Effect of Fe-containing supports prepared by a novel sol-gel method in the CO methanation reaction: CO elimination and synthetic natural gas production. Energy Technol..

[18] Wang S., Liu C., Liu G., Zhang M., Li J., Wang C. (2011). Fabrication of superhydrophobic wood surface by a sol-gel process. Appl. Surf. Sci..

[19] McKeen L.W. (2006). Fluorinated Coatings and Finishes Handbook: The Definitive User’s Guide.

[20] Textor T., Mahltig B. (2010). A sol-gel based surface treatment for preparation of water repellent antistatic textiles. Appl. Surf. Sci..

[21] Arkles B. (2006). Hydrophobicity, hydrophilicity, and silanes. Paint Coat. Ind..

[22] Hsieh C.T., Chen J.M., Huang Y.H., Kuo R.R., Li C.T., Shih H.C., Lin T.S., Wu C.F. (2006). Influence of fluorine/carbon atomic ratio on superhydrophobic behavior of carbon nanofiber arrays. J. Vac. Sci. Technol. B.

[23] Wang H., Fang J., Cheng T., Ding J., Qu L., Dai L., Wang X., Lin T. (2008). One-step coating of fluoro-containing silica nanoparticles for universal generation of surface superhydrophobicity. Chem. Commun..

[24] Xue C.H., Jia S.T., Zhang J., Tian L.Q. (2009). Superhydrophobic surfaces on cotton textiles by complex coating of silica nanoparticles and hydrophobization. Thin Solid Film..

[25] Wang X., Chai Y., Liu J. (2013). Formation of highly hydrophobic wood surfaces using silica nanoparticles modified with long-chain alkylsilane. Holzforschung.

[26] Stober W., Fink A., Bohn E. (1968). Controlled growth of monodisperse silica spheres in the micron size range. J. Colloid Interface Sci..

[27] Gurav A.B., Latthe S.S., Vhatkar R.S. (2013). Sol-gel-processed porous water-repellent silica microbowls. Surf. Innov..

[28] Tombesi A., Li S., Sathasivam S., Page K., Heale F.L., Pettinari C., Carmalt C.J., Parkin I.P. (2019). Aerosol-assisted chemical vapour deposition of transparent superhydrophobic film by using mixed functional alkoxysilane. Sci. Rep..

[29] Yu C., Wang F., Lucia L.A., Fu S. (2017). Induction of superhydrophobicity in a cellulose substrate by LbL assembly of covalently linked dual-sized silica nanoparticle layers. Adv. Mater. Phys. Chem..

[30] Wang X., Li X., Lei Q., Wu Y., Li W. (2018). Fabrication of superhydrophobic composite coating based on fluorosilicone resin and silica nanoparticles. R. Soc. Open Sci..

[31] Kanimozhi M. (2011). Investigating the physical characteristics of *Sansevieria trifasciata* Fibre. Int. J. Sci. Res. Publ..

[32] Perera H.J., Goyal A., Alhassan S.M. (2022). Surface properties of alkylsilane treated date palm fibre. Sci. Rep..

[33] Morshed M., Alam M.M., Daniels S. (2010). Plasma treatment of natural jute fibre by RIE plus plasma tool. Plasma Sci. Technol..

[34] Rajeshkumar G. (2020). Characterization of surface modified *Phoenix* sp. fbers for composite reinforcement. J. Nat. Fibers.

[35] Kabir M.M., Wang H., Cardona F., Aravinthan T. (2010). Effect of chemical treatment on the mechanical and thermal properties hemp fibre reinforced thermoset sandwich composites. Inc. Sustain. Pract. Mech. Struct. Mater..

[36] Chen H., Zhang W., Wang X., Wang H., Wu Y., Zhong T., Fei B. (2018). Effect of alkali treatment on wettability and thermal stability of individual bamboo fibers. J. Wood Sci..

[37] Mittal M., Chaudhary R. (2019). Experimental investigation on the thermal behaviour of untreated and alkali-treated pineapple leaf and coconut husk fibers. Int. J. Appl. Sci. Eng..

[38] Reddy G.R., Kumar M.A., Jayaramudu J. (2014). Biodegradable *Sansevieria cylindrica* leaf fiber/Tamarind fruit fiber based polymer hybrid composites on characterizations. Int. Lett. Chem. Phys. Astronony.

[39] Hebbar R.S., Isloor A.M., Ismail A.F. (2017). Contact angle measurements. Membr. Charact..

[40] Peng L., Qisui W., Xi L., Zhang C. (2009). Investigation of the states of water and OH groups on the surface of silica. Colloids Surf. A Physicochem. Eng. Asp..

[41] Jose J.P., Malhotra S.K., Thomas S., Joseph K., Goda K., Sreekala M.S. (2012). Advances in polymer composites: Macro- and microcomposites—State of the art, new challenges, and opportunities. Polym. Compos..

[42] Masuelli M.A. (2013). Introduction of fibre-reinforced polymers–polymers and composites: Concepts, properties and processes. Fiber Reinforced Polymers: The Technology Applied for Concrete Repair.

[43] Ali A., Shaker K., Nawab Y., Ashraf M., Basit A., Shahid S., Umair M. (2015). Impact of hydrophobic treatment of jute on moisture regain and mechanical properties of composite material. J. Reinf. Plast. Compos..

[44] Doleza P.I., Arfaouia M.A., Dubéb M., David E. (2017). Hydrophobic treatments for natural fibers based on metal oxide nanoparticles and fatty acids. Procedia Eng..

[45] Jiang X., Li Q., Li X., Meng Y., Ling Z., Ji Z., Chen F. (2022). Preparation and characterization of degradable cellulose based paper with superhydrophobic, antibacterial, and barrier properties for food packaging. Int. J. Mol. Sci..

